# Factors Predictive of Positive Lymph Nodes for Breast Cancer

**DOI:** 10.3390/curroncol30120754

**Published:** 2023-12-06

**Authors:** Kelly M. Elleson, Katherine Englander, Julia Gallagher, Neha Chintapally, Weihong Sun, Junmin Whiting, Melissa Mallory, John Kiluk, Susan Hoover, Nazanin Khakpour, Brian Czerniecki, Christine Laronga, Marie Catherine Lee

**Affiliations:** 1 Regional Breast Care, Genesis Care Network, 8931 Colonial Center Dr #301, Fort Myers, FL 33905, USA; 2 Morsani College of Medicine, University of South Florida, Tampa, Fl 33602, USAnehac@usf.edu (N.C.); 3 Department of Breast Oncology, H. Lee Moffitt Cancer Center and Research Institute, Tampa, FL 33612, USAchristine.laronga@moffitt.org (C.L.); 4 Department of Biostatistics and Bioinformatics, H. Lee Moffitt Cancer Center and Research Institute, Tampa, FL 33612, USA

**Keywords:** breast cancer, sentinel lymph node, surgery, lymph node metastases

## Abstract

Background: Axillary node status is an important prognostic factor in breast cancer. The primary aim was to evaluate tumor size and other characteristics relative to axillary disease. Materials and Methods: Single institution retrospective chart review of stage I-III breast cancer patients collected demographic and clinical/pathologic data from 1998–2019. Student’s *t*-test, Chi-squared test (or Fisher exact test if applicable), and logistic regression models were used for testing the association of pN+ to predictive variables. Results: Of 728 patients (mean age 59 yrs) with mean follow up of 50 months, 86% were estrogen receptor +, 10% Her2+, 78% ER+HER2−negative, and 10% triple-negative. In total, 351/728 (48.2%) were pN+ and mean tumor size was larger in pN+ cases compared to pN− cases (mean = 27.7 mm versus 15.5 mm) (*p* < 0.001). By univariate analysis, pN+ was associated with lymphovascular invasion (LVI), higher grade, Her2, and histology (*p* < 0.005). Tumor-to-nipple distance was shorter in pN+ compared to pN− (45 mm v. 62 mm; *p*< 0.001). Age < 60, LVI, recurrence, mastectomy, larger tumor size, and shorter tumor-nipple distance were associated with 3+ positive nodes (*p* < 0.05). Conclusions: Larger tumor size and shorter tumor-nipple distance were associated with higher lymph node positivity. Age less than 60, LVI, recurrence, mastectomy, larger tumor size, and shorter tumor-nipple distance were all associated with 3+ positive lymph nodes.

## 1. Introduction

Breast cancer is currently the most diagnosed cancer in women worldwide with an estimated 2.3 million new cases in 2020 [1]. The presence of metastatic disease in axillary lymph nodes is one of the most important prognostic variables impacting breast cancer treatment and survival. Preoperative evaluation of axillary lymph nodes is variable across practices due to the availability of technology and national or international guidelines. Axillary assessment ranges from a basic clinical physical exam to radiographic studies, typically with ultrasound, or less commonly, magnetic resonance imaging (MRI), and possibly percutaneous biopsy. The ability to diagnose abnormal lymph nodes on physical exam does have limitations, with up to 33–53% of “clinically node negative” patients having positive lymph nodes on histopathology [2,3]. In patients with a clinically negative axilla, the gold standard to determine lymph node status is the sentinel lymph node biopsy. Sentinel lymph node biopsy is usually performed at the time of tumor excision and serves to identify the first lymph nodes that drain the breast, utilizing a radiotracer and/or blue dye. Axillary lymph node metastasis guides further treatment, including additional surgical excision with axillary lymph node dissection, radiation therapy, and systemic treatments such as chemotherapy. 

Systemic treatments are also driven by the breast cancer subtype, typically broken down by expression of human epidermal growth factor receptor 2 (HER2) and hormone receptors, consisting of estrogen receptors (ER) and progesterone receptors (PR). The majority (70%) of breast cancer in western populations is ER+, while 10–15% overexpress HER2 (Her2+), and 20% lack all three receptors, known as triple-negative breast cancer [4]. 

Over the years, axillary surgery has diminished significantly; this is a multifactorial phenomenon. The strongest rationales for moving away from extensive axillary surgery are the long-term morbidities associated with axillary clearance as well as the lack of survival benefit. Axillary node dissection is associated with lymphedema, which is reported in 20–40% of patients undergoing axillary dissection. Lymphedema is variable both in timing as well as extent, presenting in the immediate postoperative period or even years after lymph node clearance, and can range from a minor intermittent nuisance to progressive and disabling limb swelling. Less visible but equally prevalent is the known morbidity of chronic neuropathy, numbness, or neuropathic pain in the axillary region. Given that axillary clearance is not associated with improved overall survival, and that systemic therapy continues to improve, the rationale for extensive axillary surgery continues to weaken, with increasing emphasis on less invasive or even noninvasive axillary evaluation.

One of the strongest drivers away from axillary dissection is the impact of screening mammography in the detection of breast cancer, with a majority of American women presenting with early-stage breast cancer detected on screening mammography, rather than presenting with palpable breast or axillary masses. Most of these women will not have symptomatic axillary metastases, so there is no therapeutic benefit to axillary dissection in this population. Because of early detection, axillary surgery evolved away from universal axillary lymph node dissection to the more selective diagnostic sampling of sentinel lymph node biopsy in the 1990s, with a significant decrease in the incidence of lymphedema and neuropathy. 

Even more recently, omission of lymph node surgery entirely in the elderly low-risk population or even well-selected early breast cancers [5] has been a focus of investigation. With a growing emphasis on gene expression as a predictor of outcome, the actual impact of sentinel node biopsy status has decreased, particularly for hormone-responsive early breast cancer, where endocrine therapy is the backbone of systemic therapy and the addition of traditional chemotherapy often does not confer a significant survival advantage. With this in mind, the Society of Surgical Oncology 2016 Choosing Wisely guideline recommended the omission of sentinel node biopsy for T2 or smaller hormone receptor-positive Her2−negative breast cancers in women over 70; this has been widely adopted in many institutions in the United States [6,7]. Even more notable are the results of the long-awaited SOUND trial, which boldly suggests the omission of axillary staging in small, hormone responsive, Her2−negative breast cancers if no ipsilateral sonographic adenopathy is detected at presentation.

De-escalation of axillary surgery has even been supported in node-positive women undergoing breast conserving surgery with low volume, pathologically identified axillary metastases [8], as well as those who are down staged after neoadjuvant chemotherapy. Due to improvements in systemic therapy, avoidance of an axillary node dissection has been described in 50–80% of triple-negative and Her2−positive cancers [9]. Preoperative imaging of the axilla, most commonly ultrasound, or MRI may provide additional information to the clinician to help guide the above decision making, with preoperative ultrasound having an accuracy of 88.7% [10]. However, there are currently no consensus recommendations for routine imaging evaluation of the axilla in clinically node-negative breast cancer patients. The primary goal of our study was to determine if certain clinical factors such as tumor size, location, and receptor subtype are predictive of metastatic disease to lymph nodes in clinically node-negative breast cancer patients. 

## 2. Methods

### 2.1. Patient Selection

After obtaining Institutional Review Board approval, we performed a single institution, retrospective review of female patients with their first diagnosis of unilateral invasive stage I-III breast cancer from 1998 to 2019. We identified patients that underwent upfront surgery via mastectomy or lumpectomy. All patients were clinically node-negative at presentation. Excluded were women who received neoadjuvant chemotherapy, or had multicentric, bilateral, inflammatory, and in situ-only disease. The study cohort was identified from a prospectively collected single institution retrospective database of breast cancer patients treated at our NCI-designated comprehensive cancer center from 2000 to 2022. The medical record was reviewed to collect demographic information, pathology, treatment, recurrence, and survival data. Tumors were restaged according to the AJCC 7th edition staging manual. Tumor locations were obtained by review of imaging records and classified as upper outer, lower outer, upper inner, lower inner, axillary tail, and central/retroareolar locations identified by the clock face of the tumor location and classified on chart review. Tumor distance from the nipple was obtained from imaging records measuring the distance of the biopsy-proven lesion to the nipple; receptor status/breast cancer subtype was identified by review of the initial core biopsy pathology reports.

### 2.2. Statistical Analysis

We performed a subset analysis of patients based on molecular subtype. Descriptive statistics (frequencies and proportions for categorical variables; mean, standard deviation, median and ranges for continuous variables) were used to summarize patient characteristics. Student’s *t*-test was used to assess the association of continuous variables and lymph node positivity. Chi-squared test (or Fisher exact test if applicable) was used to assess the association of categorical variables and lymph node positivity. The Univariate/Multivariate logistic regression model was applied to determine the impact of each predictive variable on the odds ratio of lymph node positivity, or 3 or more positive lymph nodes. Multivariate model development was completed by first including variables with *p* < 0.25 into an initial model, followed by backward elimination to remove variables with *p* > 0.05 from the final model. All statistical tests were two-sided, with the level of significance established at *p* < 0.05. All analyses were performed using SAS (Version 9.4, SAS Institute Inc., Cary, NC, USA). 

## 3. Results

### 3.1. Evaluation of All Patients 

Seven hundred twenty-eight female patients between the ages of 21–96 years (median age 61 years) old were identified with primary invasive breast cancer diagnosed between 1998 and 2019 and treated with upfront surgery. Four hundred (54.9%) women underwent breast conservation surgery, while three hundred twenty-eight (45.1%) underwent a mastectomy. Most tumors were T1c (40.8%), invasive ductal carcinoma (IDC; 84.1%), histologic grade 2 (49.9%), without lymphovascular invasion (81%), estrogen (ER; 86.1%), progesterone receptor-positive (PR;80.2%), and without HER2 overexpression (88.5%) (Table 1). Median follow-up was 40.6 months (range 0.3–242 months). (Table 1).

All patients were clinically node-negative with the majority of women undergoing a sentinel lymph node biopsy (99.9%), with 48.2% having at least one positive lymph node, of which 231/351 (65.8%) women underwent an axillary lymph node dissection. The median nodes obtained during an axillary dissection was 15 (range 1–43) with a median of 0 additional positive nodes (range 0–20 nodes). 

**Figure 1:**
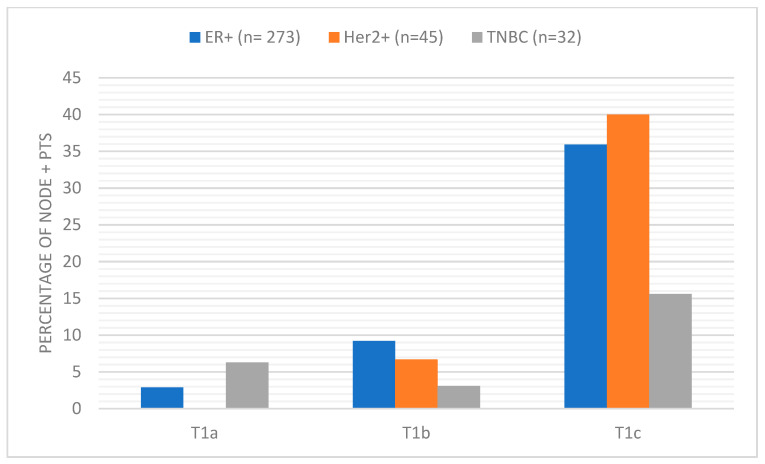
Percentage of node-positive patients broken down by T size and receptor subtype.

Women with larger size tumors were more likely to have positive axillary lymph nodes (*p* < 0.001) and subsequently undergo an axillary lymph node dissection (mean 17.6 mm vs. 29.6 mm; *p* < 0.001) (Figure 1. Patients with positive axillary lymph nodes had a mean tumor size of 27.7 mm, while those with negative axillary lymph nodes had a mean tumor size of 15.5 mm (*p* < 0.001). However, tumor size was not associated with the quantity of positive axillary lymph nodes. 

Patients with positive axillary lymph nodes had a shorter tumor-to-nipple distance of 45.0 mm compared to patients with negative axillary lymph nodes with a tumor-to-nipple distance of 62.1 mm (*p* < 0.001). Two hundred sixty-four patients (36.7%) had tumors located in the upper outer quadrant, while 203 (28.2%) had tumors in overlapping quadrants. Tumor location within the breast was not associated with lymph node positivity; however, on univariate logistic regression analysis of patients with positive lymph nodes, tumors located near the nipple–areolar complex had an odd ratio (OR) of 2.00 (96%CI 1.05–3.82; *p* = 0.036). On univariate logistic regression analysis, grade (*p* < 0.001), tumor size (*p* < 0.001), histology (*p* < 0.001), Her2+ (*p* = 0.020), lymphovascular invasion (LVI; *p* < 0.001), and age (*p* < 0.001) at diagnosis were all associated with axillary lymph node positivity (Table 2). On multivariable logistic regression, larger tumor size (*p* < 0.001), younger age at diagnosis (*p* = 0.005), shorter tumor-to-nipple distance (*p* < 0.001), and surgery type, in particular mastectomy (*p* < 0.001), were all associated with axillary lymph node positivity. 

Further univariant analysis found lymphovascular invasion, recurrence, and age at diagnosis less than 60 years old to be associated with three or more positive lymph nodes (*p* < 0.004). Median tumor size of patients with three or more positive lymph nodes was 26 mm, while patients with 0–2 positive lymph nodes had a median tumor size of 16 mm (*p* < 0.001). Similarly, median tumor-to-nipple distance was shorter (40 mm) in patients with three or more positive lymph nodes compared to 0–2 positive lymph nodes (60 mm; *p* = 0.009). Receptor status had no significant association with three or more positive lymph nodes. (Table 3) Multivariate analysis found lymphovascular invasion presence (OR 2.51; *p* = 0.007) and mastectomy (OR 3.30; *p* = 0.005) to be associated with three or more positive lymph nodes. 

### 3.2. Subset Evaluation of ER+

Five hundred and sixty-seven patients within the cohort were diagnosed with estrogen receptor-positive (ER+), Her2−negative breast cancer. The median age of diagnosis was 62 years (range 30–96 years) with a median follow-up period of 39.9 months (range 0.3–200 months). Within the median follow-up time, twenty-eight patients had distant recurrence, three had local recurrence, and six patients had locoregional recurrence. At the time of last follow-up, thirty-five patients were deceased due to breast cancer. Most tumors were T1 (64.7%), histologic grade 2 (57.6%), and invasive ductal carcinoma (81.1%). Additionally, 76 patients had extranodal extension, and 75 patients had lymphovascular invasion. 

All patients underwent a sentinel lymph node biopsy, and one hundred sixty-eight patients had an axillary lymph node dissection. Of the five hundred sixty-seven patients with estrogen receptor-positive tumors, 272 (48.0%) patients were lymph node-positive, including micrometastatic nodal involvement. Patients with node-positive disease had a significantly larger primary tumor size (mean = 28.4 mm) compared to their node-negative counterparts (mean = 15.6 mm) (*p* < 0.001). However, total nodal burden was not significantly associated with primary tumor size (*p* = 0.793) or tumor-to-nipple distance (*p* = 0.693). The proportion of node-positive patients increased with increasing T stage between T1mi and T2 (*p* < 0.001). The highest number of tumors were in the upper-outer quadrant (UOQ) (*n* = 210); however, tumor quadrant location was not significantly associated with lymph node status (*p* = 0.145).

### 3.3. Subset Evaluation of Triple-Negtaive Breast Cancer (TNBC)

We identified seventy-three patients diagnosed with TNBC who had a median age of 56 years (range 21–85 years). Most tumors (42.5%) were T2 stage at presentation, 25.9% of patients had lymphovascular invasion, 17.8% had recurrence of TNBC, and 13.7% patients died due to cancer-related mortality by the end of the follow-up period (mean 48 months).

On surgical pathology, 43.8% of TNBC patients were node-positive on sentinel node biopsy. Mean tumor size was 20 mm (range 1–53 mm). Node-positive patients had a larger average tumor size of 27.3 mm compared to 14.3 mm for node-negative patients (*p* < 0.001). T stage was also found to be significantly associated with sentinel lymph node positivity (*p* < 0.001) [11].

The mean tumor-to-nipple distance as detected by ultrasound was 65.5 mm (range 10–140 mm). We found a higher likelihood of sentinel lymph node positivity among patients with shorter tumor-to-nipple distance (*p* = 0.005). The average distance to the nipple was 51.0 mm for SLN-positive patients compared to 73.3 mm for node-negative patients. NMost patients (33/73) had primary tumors located in the axillary tail/upper outer quadrant; however, no significant association was found between tumor quadrant and sentinel lymph node positivity (*p* = 0.653). 

### 3.4. Subset Evaluation of Her2+

**Figure 2:**
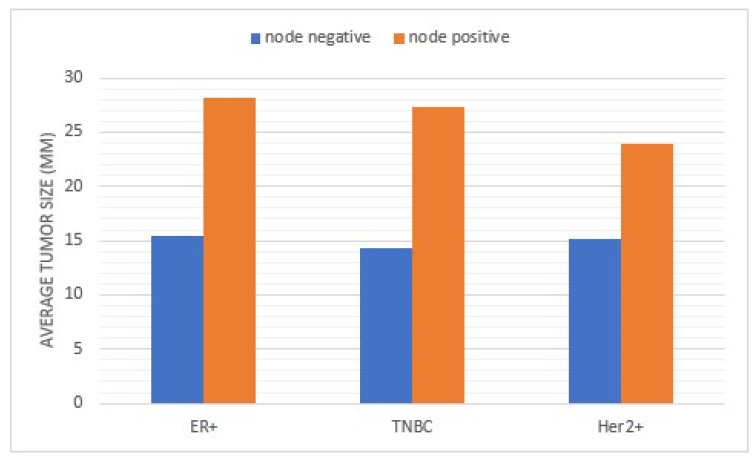
Comparison of average tumor size for lymph node-positive and negative patients broken down by molecular subtype.

Seventy-five of 728 (10.3%) patients had Her2+ disease. The mean age at diagnosis was 53.5 (range 21–85). IDC was the most common diagnosis (97.3%) and most patients had T1 disease (62.7%) [12] Most patients had histologic grade 3 (62.7%) disease, and 20% had lymphovascular invasion. Out of 47 T1 patients, 21 were positive for nodal metastasis (44.7%), a larger percentage compared to other subtypes (Figure 2). Breast cancer recurrence was observed in 18.7% of patients at the end of the follow-up period (mean 65.9 months). Seven patients had distant recurrence, five had local recurrence, and two had local–regional recurrence. Patients with positive nodes had a larger mean tumor size (*n* = 45; 23.9 mm) (*p* = 0.002) than patients with negative nodes (*n* = 30; 15.1 mm). 

All seventy-five HER2+ patients had a sentinel lymph node biopsy. The mean number of positive nodes on sentinel lymph node biopsy was 2.4 (range 1–9). Axillary lymph node dissection was performed in thirty-seven cases, which found a mean of 1.4 positive nodes. LVI was significantly associated with larger tumor size (29.6 mm v 20.3 mm; *p* = 0.010). There was a significant relationship between larger tumor size and surgical treatment with mastectomy (24.5 mm) compared to lumpectomy (16.8 mm) (*p* = 0.006). 

## 4. Discussion

In the past, axillary lymph node dissection was performed on all breast cancer patients as both a staging and therapeutic intervention until the advent of the sentinel lymph node biopsy, which is a much less morbid intervention for patients presenting clinically without evidence of axillary involvement [13]. Axillary node metastases is a known poor prognostic indicator with a 28–40% decrease in 5-year survival compared to node-negative patients [14,15,16]. With the de-escalation of surgery in the axilla over the years, now more than ever, it is important to better predict preoperatively which patients have a higher chance of nodal involvement. 

Our study sought to evaluate tumor characteristics that predict axillary lymph node metastases in clinically node-negative breast cancer patients receiving upfront surgery. We reviewed patients that received surgical nodal evaluation and analyzed clinically quantifiable factors that may help predict nodal involvement with breast cancer such as tumor size, tumor distance to the nipple, and location of the tumor within the breast. Subset analysis was also performed of ER+, Her2+, and triple-negative breast cancer. Similar to other studies, we found that tumor size, grade, receptor status, and lymphovascular invasion were associated with axillary nodal involvement [17]. Lymphovascular invasion is a known marker for worse survival outcomes in breast cancer, as well as a risk factor for local recurrence and distant metastases. Lymphovascular invasion is more commonly seen in larger tumors, Her2+, and TNBC subtypes [18]. In addition, we were able to associate node-positive disease with shorter tumor-to-nipple distance and smaller tumors in the Her2+ subset compared to others. 

Previous studies have concluded that larger tumor size increases risk for axillary nodal involvement; however, increasing tumor size did not proportionally correspond to the risk of being node-positive in the overall population as well as the ER+ subset [19,20]. We found similar results in that node-positive tumors were 1.79 times larger than node-negative tumors. Molecular subtype did impact average tumor size for node-positive patients. Patients presenting with Her2+ tumors and found to have axillary nodal involvement had an average tumor size of 23.9 mm compared to ER+ tumors at 28.4 mm and TNBC lesions at 27.3 mm (*p* = 0.002). However, when looking at the number of positive lymph nodes, tumor size was not associated with nodal disease burden; this is especially relevant in a post Z-11 era, where a positive sentinel node biopsy does not commit a patient to an axillary dissection [8]. This could translate to the neoadjuvant setting, supporting the idea that tumor size alone should not be an exclusion criterion for a targeted axillary node dissection to evaluate for pathologic complete response and avoid axillary node dissection. 

Most of our study population consisted of smaller clinically node-negative ER+Her2− breast cancers, which is the most common clinical scenario in the general United States breast cancer population. With this population, the preoperative question of which patients have positive axillary lymph nodes has shifted to which patients have three or more positive axillary nodes following Z-11 [8]. Our study found that age less than 60, lymphovascular invasion, recurrence, mastectomy, larger tumor size, and shorter tumor-to-nipple distance were all associated with three or more positive lymph nodes. These risk factors for three or more positive lymph nodes would support additional imaging evaluation of the axilla in the form of an ultrasound. Preoperative knowledge of nodal positivity can facilitate nodal localization for targeted axillary dissection, neoadjuvant chemotherapy discussion, and metastatic evaluation. 

Some studies have shown that tumor proximity to the nipple is a risk factor for axillary lymph node involvement [21,22,23]. The lymphatics found under the nipple–areolar complex are different from other areas of the breast, with a denser network of lymphatic capillaries known as the subareolar plexus [24]. Our study showed that across all subtypes, axillary lymph node-positive patients had a shorter average tumor-to-nipple distance, with TNBC having the shortest distance. Tumor location at the nipple–areolar complex was also associated with node-positive disease, supporting the underlying mechanism of increased lymphatics behind the nipple. 

There are conflicting studies evaluating molecular subtype impact on axillary lymph node status. Alsumai et al. found that hormone receptor-positive Her2− tumors had a 83.7% higher likelihood of SNB positivity compared to other subtypes [17], while others demonstrated no differences [25,26]. Our study showed that patients with Her2+ tumors were more likely to have positive axillary nodes and those patients presented with smaller tumor size compared to other subtypes. In our study population, over half (62.7%) of Her2+ tumors had grade 3 tumors, which further supports the aggressive nature of the subtype. One proposed mechanism of Her2+ tumors metastasizing more often is the overexpression of vascular endothelial growth factor C, which is expressed mostly in lymphatic endothelial cells and promotes lymph node metastasis [27]. 

We recognize the inherent flaws in our retrospective study design. The greatest limitation is an inability to analyze long-term follow-up in our patient cohort. Our analysis is further hampered by missing values when evaluating certain variables. Specifically, values were missing from tumor grade, tumor-to-nipple distance, extranodal extension, lymphovascular invasion, and tumor location. Additionally, the relatively low proportions of T3 tumors, along with TNBC and Her2+ lesions, reflect an intrinsic selection bias, as a majority of these patients are treated with chemotherapy prior to surgery at our institution. Tumor heterogeneity of breast cancer provides limitations when looking at a combined analysis of HER2−positive, triple-negative tumor, and HR+HER2−negative breast cancer as biologically they have different behavior. We also recognize that micrometastatic disease is treated differently from macrometastatic disease in the axilla.

This study provides tumor characteristics that clinicians can use when planning surgical treatment upfront. With multiple risk factors present, many clinicians may want to obtain focused axillary imaging to better evaluate preoperatively which patients may be presenting with axillary lymph node metastases. High risk for axillary lymph node involvement can also impact the conversation regarding reconstruction, or on the opposite spectrum, the need for a sentinel lymph node biopsy in a geriatric patient. 

## 5. Conclusions

Larger tumor size and short tumor-nipple distance are associated with pathological nodal positivity, but not nodal disease burden. Comparing subtypes, smaller Her2+ tumors were associated with nodal positivity. Other factors predicting axillary lymph positivity include age, higher grade, Her2+, lymphovascular invasion, histology, and nipple–areolar complex location. Age less than 60, lymphovascular invasion, recurrence, mastectomy, larger tumor size, and shorter tumor-to-nipple distance were all associated with three or more positive lymph nodes. Given the wholesale migration away from axillary surgery and complete axillary clearance in particular, these preoperative risk factors can guide clinicians’ need for preoperative axillary imaging and surgical planning. 

## Figures and Tables

**Table 1:** Demographics of 728 patients evaluated.

Variable	Level	*N* = 728	%
**Grade**	1	123	17.0
	2	362	49.9
	3	239	33.0
**ER**	Negative	101	13.9
	Positive	627	86.1
**PR**	Negative	144	19.8
	Positive	584	80.2
**Her2**	Negative	644	88.5
	Positive	75	10.3
	Indeterminate	9	1.2
**ER/Her2**	ER+/Her2−	567	77.9
**ER/PR/Her2**	ER−/PR−/Her2−	73	10.0
**Histology**	IDC	612	84.1
	ILC	80	11.0
	IMC	19	2.6
	Other	17	2.3
**Alive status**	Alive	676	92.9
	Death	52	7.1
**Recurrence**	No	664	91.2
	Yes	64	8.8
**Recurrence Type (*n* = 64)**	Distant	42	65.6
	Local	10	15.6
	Locoregional	12	18.8
**Sentinel Lymph Node Biopsy**	No	1	0.1
	Yes	727	99.9
**SLNB results**	Negative	377	51.8
	Positive	350	48.2
**Axillary lymph node** **dissection**	No	497	68.3
	Yes	231	31.7
**Surgery Type**	Lumpectomy	400	54.9
	Mastectomy	328	45.1
**Stage T**	1mi	6	0.8
	1a	60	8.2
	1b	101	13.9
	1c	297	40.8
	2	234	32.1
	3	30	4.1
**LVI**	No	464	81.0
	Yes	101	17.6
	Indeterminate	8	1.4
**Tumor location**	Axillary Tail/UOQ	264	36.7
	LIQ	40	5.6
	LOQ	68	9.4
	Nipple/Areolar/Retroareolar	48	6.7
	Overlapping	203	28.2
	UIQ	97	13.5
**Age at diagnosis**	Mean	59.5	
	Median	61	
	Minimum	21	
	Maximum	96	
	Std Dev	12.2	
**Tumor size (mm)**	Mean	21.4	
	Median	17	
	Minimum	0.6	
	Maximum	320	
	Std Dev	22.4	
**# nodes obtained during ALND**	Mean	16.4	
	Median	15	
	Minimum	1	
	Maximum	43	
	Std Dev	7.4	
**# of Additional Positive Nodes in ALND**	Mean	1.3	
	Median	0	
	Minimum	0	
	Maximum	20	
	Std Dev	3.2	
**Follow-up (months)**	Mean	49.7	
	Median	40.6	
	Minimum	0.3	
	Maximum	242	

Abbreviations: ER, estrogen receptor; PR, progesterone receptor; IDC, invasive ductal carcinoma; ILC, invasive lobular carcinoma; IMC, invasive mammary carcinoma; LVI, lymphovascular invasion, #, number.

**Table 2:** Univariate logistic regression model analysis of lymph node-positive patients.

	*N*	Odds Ratio (95% CI)	Overall *p*-Value
Age at diagnosis 40–60 <40 >60	321 39 368	2.21 (1.63–3.00) 5.56 (2.56–12.05) reference	**<0.001**
Grade 1 2 3	123 362 240	reference 1.63 (1.07–2.49) 2.43 (1.55–3.82)	**<0.001**
ER+ (ref = ER−)	627	0.90 (0.59–1.37)	0.621
Her2+ (ref = Her2−)	75	1.67 (1.02–2.71)	**0.020**
LVI presence (ref = no LVI)	101	91.74 (12.69–663.18)	**<0.001**
Positive Margin (ref = negative margin)	62	2.49 (1.41–4.37)	**0.002**
Histology ILC IMC Other IDC	80 19 17 612	1.25 (0.79–2.00) 6.37 (1.84–22.07) 8.95 (2.03–39.48) reference	**<0.001**
Surgery Type Lumpectomy Mastectomy	400 328	0.14 (0.10–0.20) reference	**<0.001**
Tumor Location LIQ LOQ NAC Overlapping UIQ UOQ	40 68 48 203 97 264	0.74 (0.38–1.45) 0.94 (0.55–1.61) **2.00 (1.05–3.82)** 0.78 (0.54–1.13) 0.70 (0.44–1.12) reference	0.065

Abbreviations: ER, estrogen receptor; LVI, lymphovascular invasion; IDC, invasive ductal carcinoma; ILC, invasive lobular carcinoma; IMC, invasive mammary carcinoma.

**Table 3:** Univariant analysis of patients with 3 or more positive lymph nodes.

	0–2 Positive LNs *N* = 660 (%)	3+ Positive LNs *N* = 68 (%)	*p*-Value
Age at Diagnosis <40 40–60 >60	35 (89.7) 282 (87.9) 343 (93.2)	4 (10.3) 39 (12.1) 24 (6.8)	**0.045**
Grade 1 2 3	113 (91.9) 330 (91.2) 215 (89.6)	10 (8.1) 32 (8.8) 25 (10.4)	0.724
ER Positive Negative	565 (90.1) 95 (94.1)	62 (9.9) 6 (5.9)	0.206
Her2 Positive Negative	68 (90.7) 583 (90.5)	7 (9.3) 61 (9.5)	0.625
LVI Presence	67 (66.3)	34 (33.7)	**<0.001**
Recurrence	47 (73.4)	17 (26.6)	**<0.001**
Surgery Lumpectomy Mastectomy	390 (97.5) 270 (82.3)	10 (2.5) 58 (17.7)	**<0.001**
Median Tumor Size (mm)	16	26	**<0.001**
Median Tumor distance to Nipple (mm)	60	40	**0.009**

Abbreviations: ER, estrogen receptor; LVI, lymphovascular invasion.

## Data Availability

The data presented in this study are available on request from the corresponding author. The data are not publicly available due to patient confidentiality.

## References

[1] Sung H., Ferlay J., Siegel R., Laversanne M., Soerjomataram I., Jemal A., Bray F. (2021). Global cancer statistics 2020: GLOBOCAN estimates of incidence and mortality worldwide for 36 cancers in 185 countries. CA Cancer J. Clin..

[2] Goyal S., Jacob L.A., Lokanatha D., Babu S., Lokesh K.N., Rudresha A.H., Saldanha S., Amirtham U., Thottian A.G., Rajeev L.K. (2022). Discordance in clinical versus pathological staging in breast cancer: Are we undermining the significance of accurate preoperative staging in the present era?. Breast Dis..

[3] Plichta J.K., Thomas S.M., Sergesketter A.R., Greenup R.A., Fayanju O.M., Rosenberger L.H., Tamirisa N., Hyslop T., Hwang E.S. (2019). Clinical and pathological stage discordance among 433,514 breast cancer patients. Am. J. Surg..

[4] Łukasiewicz S., Czeczelewski M., Forma A., Baj J., Sitarz R., Stanisławek A. (2021). Breast Cancer-Epidemiology, Risk Factors, Classification, Prognostic Markers, and Current Treatment Strategies—An Updated Review. Cancers.

[5] Gentilini O.D., Botteri E., Sangalli C., Galimberti V., Porpiglia M., Agresti R., Luini A., Viale G., Cassano E., Peradze N. (2023). Sentinel Lymph Node Biopsy vs No Axillary Surgery in Patients With Small Breast Cancer and Negative Results on Ultrasonography of Axillary Lymph Nodes: The SOUND Randomized Clinical Trial. JAMA Oncol..

[6] Thompson J., Le J., Hop A., Melnik M., Paulson J., Wright G.P. (2021). Impact of Choosing Wisely Recommendations on Sentinel Lymph Node Biopsy and Postoperative Radiation Rates in Women over Age 70 Years with Hormone-Positive Breast Cancer. Ann. Surg. Oncol..

[7] Welsh J.L., Hoskin T.L., Day C.N., Habermann E.B., Goetz M.P., Boughey J.C. (2017). Predicting Nodal Positivity in Women 70 Years of Age and Older with Hormone Receptor-Positive Breast Cancer to Aid Incorporation of a Society of Surgical Oncology Choosing Wisely Guideline into Clinical Practice. Ann. Surg. Oncol..

[8] Giuliano A.E., Ballman K.V., McCall L., Beitsch P.D., Brennan M.B., Kelemen P.R., Ollila D.W., Hansen N.M., Whitworth P.W., Blumencranz P.W. (2017). Effect of Axillary Dissection vs No Axillary Dissection on 10-Year Overall Survival Among Women with Invasive Breast Cancer and Sentinel Node Metastasis: The ACOSOG Z0011 (Alliance) Randomized Clinical Trial. JAMA.

[9] Montagna G., Mamtani A., Knezevic A., Brogi E., Barrio A.V., Morrow M. (2020). Selecting Node-Positive Patients for Axillary Downstaging with Neoadjuvant Chemotherapy. Ann. Surg. Oncol..

[10] Rukanskienė D., Veikutis V., Jonaitienė E., Basevičiūtė M., Kunigiškis D., Paukštaitienė R., Čepulienė D., Poškienė L., Boguševičius A. (2020). Preoperative Axillary Ultrasound versus Sentinel Lymph Node Biopsy in Patients with Early Breast Cancer. Medicina.

[11] Chintapally N., Englander K., Gallagher J., Elleson K., Sun W., Whiting J., Laronga C., Lee M.C. (2023). Tumor Characteristics Associated with Axillary Nodal Positivity in Triple Negative Breast Cancer. Diseases.

[12] Englander K., Chintapally N., Gallagher J., Elleson K., Sun W., Whiting J., Laronga C., Lee M.C. (2023). Factors Influencing Lymph Node Positivity in HER2/neu+ Breast Cancer Patients. Curr. Oncol..

[13] Pavlista D., Dusková M., Novotný J., Zikán M., Strunová M., Freitag P., Zivný J. (2002). Complications of axillary dissection in breast carcinoma. Ceska Gynekol..

[14] Zahoor S., Haji A., Battoo A., Qurieshi M., Mir W., Shah M. (2017). Sentinel Lymph Node Biopsy in Breast Cancer: A Clinical Review and Update. J. Breast Cancer.

[15] Nemoto T., Vana J., Bedwani R.N., Baker H.W., McGregor F.H., Murphy G.P. (1980). Management and survival of female breast cancer: Results of a national survey by the American College of Surgeons. Cancer.

[16] Carter C.L., Allen C., Henson D.E. (1989). Relation of tumor size, lymph node status, and survival in 24,740 breast cancer cases. Cancer.

[17] Alsumai T.S., Alhazzaa N., Alshamrani A., Assiri S., Alhefdhi A. (2022). Factors Predicting Positive Sentinel Lymph Node Biopsy in Clinically Node-Negative Breast Cancer. Breast Cancer.

[18] Kuhn E., Gambini D., Despini L., Asnaghi D., Runza L., Ferrero S. (2023). Updates on Lymphovascular Invasion in Breast Cancer. Biomedicines.

[19] Viale G., Zurrida S., Maiorano E., Mazzarol G., Pruneri G., Paganelli G., Maisonneuve P., Veronesi U. (2005). Predicting the status of axillary sentinel lymph nodes in 4351 patients with invasive breast carcinoma treated in a single institution. Cancer.

[20] Minami S., Sakimura C., Irie J., Tokai Y., Okubo H., Ohno T. (2021). Predictive Factors among Clinicopathological Characteristics for Sentinel Lymph Node Metastasis in T1-T2 Breast Cancer. Cancer Manag. Res..

[21] Bevilacqua J., Cody III H., MacDonald K., Tan L., Borgen P., Van Zee K. (2002). A model for predicting axillary node metastases based on 2000 sentinel node procedures and tumour position. Eur. J. Surg. Oncol. (EJSO).

[22] Yoshihara E., Smeets A., Laenen A., Reynders A., Soens J., Van Ongeval C., Moerman P., Paridaens R., Wildiers H., Neven P. (2013). Predictors of axillary lymph node metastases in early breast cancer and their applicability in clinical practice. Breast.

[23] Voogd A.C., Coebergh J.W., Repelaer van Driel O.J., Roumen R.M., van Beek M.W., Vreugdenhil A., Crommelin M.A. (2000). The risk of nodal metastases in breast cancer patients with clinically negative lymph nodes: A population-based analysis. Breast Cancer Res. Treat..

[24] Suami H., Pan W.-R., Mann G.B., Taylor G.I. (2008). The Lymphatic Anatomy of the Breast and its Implications for Sentinel Lymph Node Biopsy: A Human Cadaver Study. Ann. Surg. Oncol..

[25] Chua B., Ung O., Taylor R., Boyages J. (2001). Frequency and predictors of axillary lymph node metastases in invasive breast cancer. ANZ J. Surg..

[26] Ding J., Jiang L., Wu W. (2017). Predictive Value of Clinicopathological Characteristics for Sentinel Lymph Node Metastasis in Early Breast Cancer. Med. Sci. Monit..

[27] Tong Z.J., Shi N.Y., Zhang Z.J., Yuan X.D., Hong X.M. (2017). Expression and prognostic value of HER-2/neu in primary breast cancer with sentinel lymph node metastasis. Biosci. Rep..

